# Tularemia presenting as suspected necrotic arachnidism

**DOI:** 10.1002/ccr3.882

**Published:** 2017-03-06

**Authors:** Heather F. Sateia, Michael T. Melia, Joseph Cofrancesco

**Affiliations:** ^1^ Division of General Internal MedicineDepartment of MedicineJohns Hopkins University School of MedicineBaltimoreMarylandUSA; ^2^ Division of Infectious DiseaseDepartment of MedicineJohns Hopkins University School of MedicineBaltimoreMarylandUSA

**Keywords:** Arachnidism, necrotic skin lesion, spider bite, splenomegaly, tick bite, tularemia

## Abstract

The true danger of the spider bite stems from misdiagnosis and resultant delay in proper treatment of entities that, unlike spider bites, are not self‐limited. Obtaining a complete exposure and travel history is central to the development of an accurate and appropriate differential diagnosis.

## Introduction

In outpatient settings, patients often present with skin lesions suspected to be from spider bites. When from a brown recluse spider (i.e., loxosceles reclusa), it is termed loxocelism. However, with no definitive diagnostic test, providers must rely on clinical acumen. The majority of spider bites are self‐limited and self‐healing, and require no therapy beyond routine wound care. For this reason, one could posit that the most dangerous complication of a “spider bite” is missing the diagnosis of another more threatening entity. This occurred during the anthrax attacks of 2001, when an afflicted child was misdiagnosed with a spider bite thereby delaying appropriate therapy for cutaneous anthrax 1. Today, the more common clinical scenario would be delayed diagnosis of staphylococcal skin infections, Stevens–Johnson Syndrome, vasculitis, or tick‐borne illnesses, among others.

One readily available, but often overlooked, piece of evidence vital to diagnosis is geography. Spiders that cause necrotic skin lesions are few; in the United States only the genus loxosceles (e.g., brown recluse) is capable. These spiders have a limited habitat and are rarely identified outside of their endemic area 2. In the United States, loxosceles can be found in the Midwest and along the border with Mexico. Loxosceles can also be found in Mediterranean and North African countries.

In the case presented below, alternative diagnoses, in addition to spider bite, should be considered as the patient lived in an area where brown recluse spiders are not endemic. Once all of the patient's symptoms were considered together as stemming from a single process, the diagnosis of tularemia could be entertained and tested. Ultimately, this may have resulted in more timely and appropriate antimicrobial therapy for a virulent pathogen.

## Case Presentation

**Figure 1:**
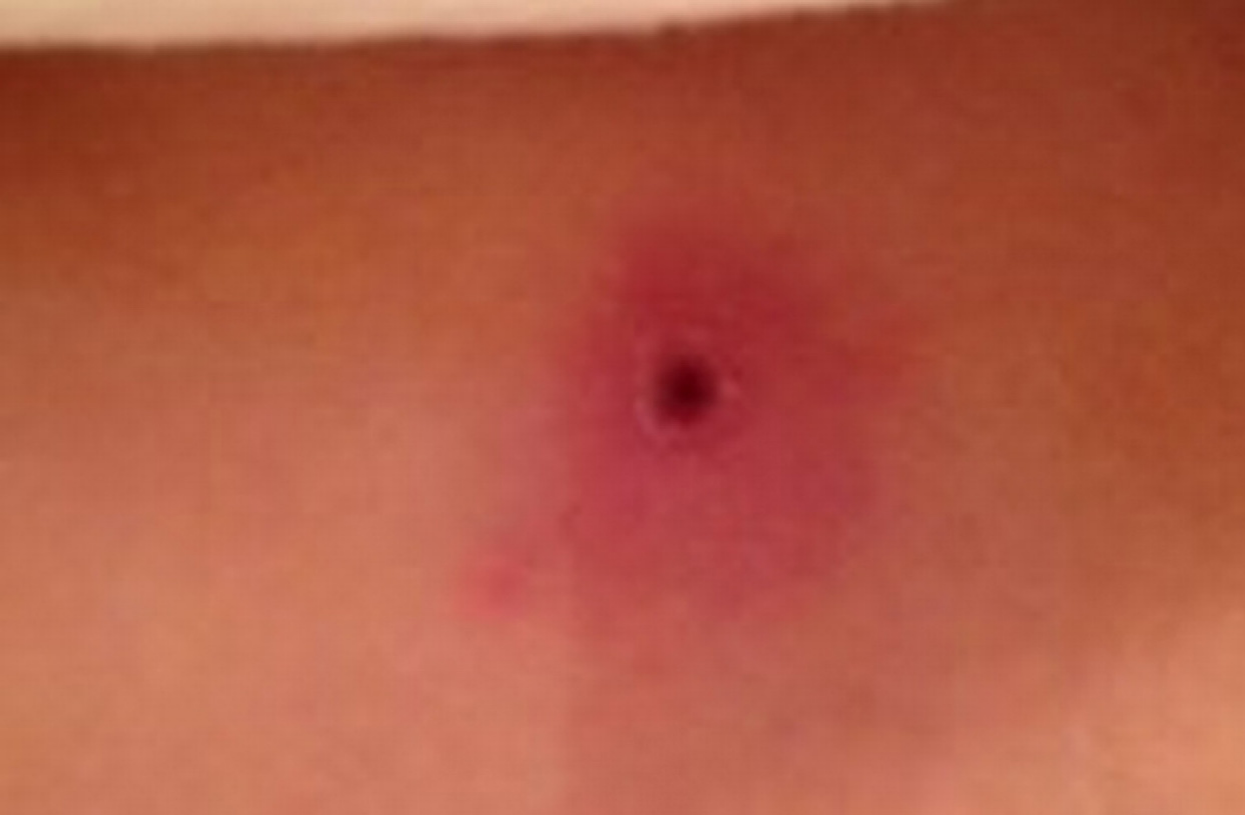
Wound at 5 days.

The patient is a 30‐year‐old female who presented to her primary care provider with a 3 × 3 cm lump behind her knee in addition to fever (103°F), chills, night sweats, and myalgia. The lesion was described as initially having been erythematous and raised with a central erosion (see Fig. 1). It was diagnosed as a spider bite and was treated with cephalexin followed by trimethoprim/sulfamethoxazole and then clindamycin, all without improvement. Despite this sequential antibiotic therapy, the patient began to experience thick, bloody drainage from the ulcerated lesion and was admitted to her local hospital for management of suspected cellulitis. A CT scan showed no abscess. While inpatient, she remained afebrile after a single dose of levofloxacin in the emergency room. The patient was discharged home without antibiotic therapy.

During a subsequent outpatient evaluation, the diagnosis of a spider bite, likely from a brown recluse was felt most likely.

**Figure 2:**
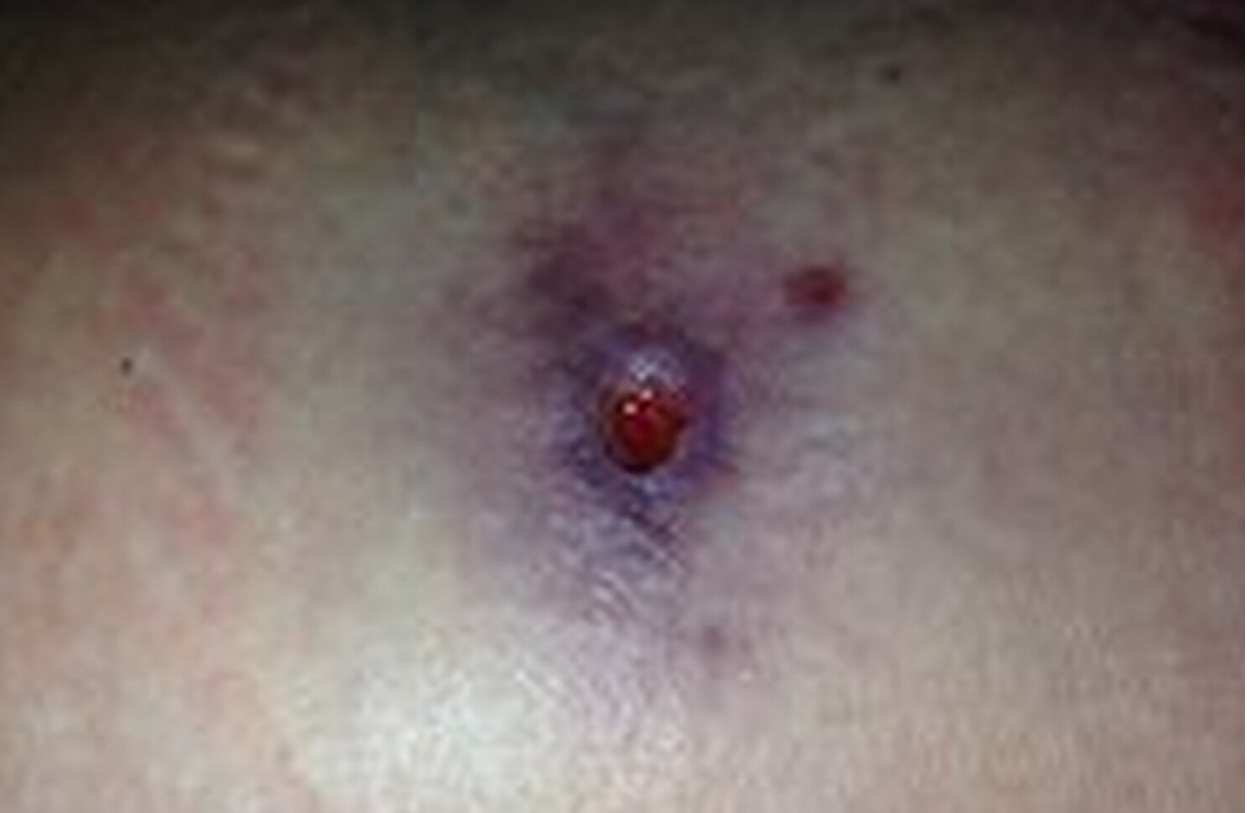
Wound at 3 days.

**Figure 3:**
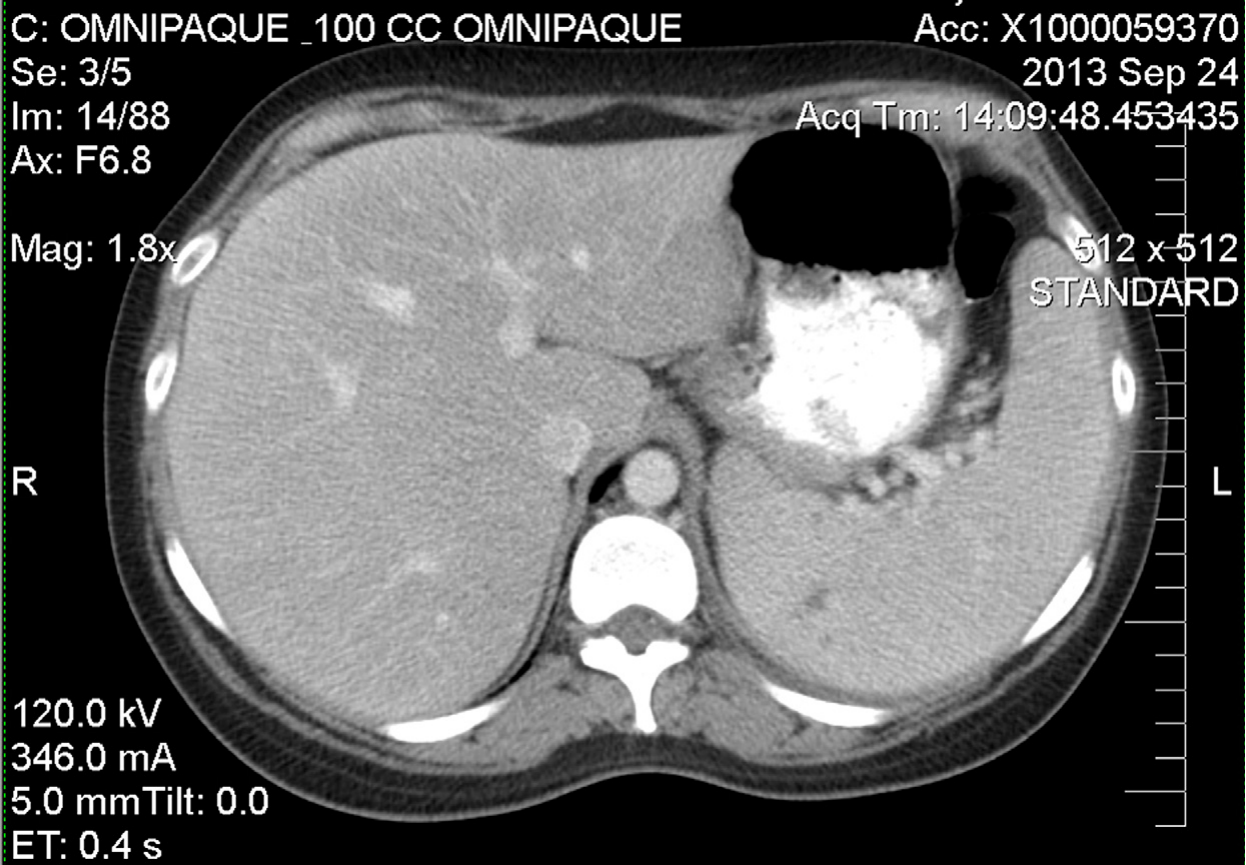
Abdominal CT showing splenomegaly.

Shortly thereafter, the patient developed sharp, stabbing left upper quadrant abdominal pain that worsened with inspiration. She was found to have an enlarged spleen on palpation; a subsequent CT confirmed splenomegaly but showed no associated lymphadenopathy (see Fig. 2). The patient was quickly referred to oncology for evaluation. Prior to seeing an oncologist, however, the patient sought a second opinion at our internal medicine practice. At the time of her visit, she endorsed a persistent lower extremity wound (see Fig. 3), progressive abdominal pain, dyspnea, an 8 kg weight loss, and severe fatigue. She had become so ill that she could not lift her one‐year‐old daughter.

Her medical history includes hypothyroidism, fibromyalgia, and depression.

She is married with a one‐year‐old daughter. She lives in rural Western Maryland and has not traveled outside of this area in over a year. She drinks alcohol very rarely and denies recent tobacco and illicit drug use. She denies unusual exposures. They have two small dogs at home but no cats. Her family history is unremarkable, including no family history of cancer.

## Investigations

Initial investigations focused on the patient's symptomatic splenomegaly, which raised concern for etiologies requiring rapid diagnosis and treatment.

Laboratories obtained upon initial outpatient evaluation were notable for mild anemia (hemoglobin 11.6 g/dL), normal white blood cell count of 7.5 thousand/*μ*L, normal lactate dehydrogenase of 177 U/L, and normal peripheral blood smear.

Her heterophile antibody screen (Monospot) was negative and Epstein–Barr virus serologies, including a positive EBNA IgG and a negative EBV VCA IgM, were consistent with remote infection. CMV IgG and IgM and HIV 1/2 antibody tests were negative. A viral hepatitis panel (including hepatitis A IgM, hepatitis B core IgM, hepatitis C antibody) was nonreactive and hepatitis B surface Ag was negative.

PA and lateral chest X‐rays showed elevation of the left hemidiaphragm and associated atelectasis at the left lung base.

After initial laboratory results were unrevealing, an investigation for tick‐borne illnesses was pursued in an effort to account for the ulcerating skin lesion, the patient's symptoms, and her splenomegaly. *Borrelia burgdorferi* ELISA and *Babesia microti*,* Anaplasma phagocytophilum*, and *Rickettsia rickettsii* IgG and IgM antibodies were negative. *Ehrlichia chaffeensis* DNA, *Ehrlichia ewingii* DNA, and *Anaplasma phagocytophilum* DNA were also negative.

*Franciscella tularensis* antibody, measured using direct agglutination, was markedly positive at 1:1280. While no convalescent specimen was available, this remarkably high titer was felt to be sufficiently elevated to substantiate a diagnosis of tularemia.

## Differential Diagnosis

Initially, the differential diagnosis for this patient centered on splenomegaly. Broad categories of hematologic, infectious, congestive, inflammatory, and infiltrative were considered 1. Within these categories, the most likely etiologies were felt to be lymphoma/leukemia, HIV infection, infectious mononucleosis, sarcoidosis, or rheumatoid arthritis. Once these entities were ruled out, the differential diagnosis was expanded to incorporate her ulcerating, necrotic skin lesion. While necrotic arachnidism is included on the differential, residence within or travel to the natural habitat of the brown recluse spider is required. Other diagnostic considerations for an ulcerated skin lesion include staphylococcal skin infections, varicella zoster, herpes simplex, syphilis (although more often located on mucosal surfaces), tick‐borne illnesses (Lyme, Rickettsia, and tularemia), mycobacterial infections, sporotrichosis, leishmaniasis, endemic fungal infections, anthrax, vasculitis, squamous cell carcinoma, Stevens–Johnson syndrome, pyoderma gangrenosum, and erythema nodosum. Of these, very few are associated with splenomegaly. Specifically, syphilis and tularemia have been reported to have some association with splenomegaly, albeit a rare finding in most cases. By including the patient's splenomegaly and skin lesion in our differential, and seeking a unifying explanation for both, we were able to reach a diagnosis.

## Treatment

Once the patient's *F. tularensis* antibody returned positive, treatment with ciprofloxacin was initiated. In this case, the patient was well enough to be treated with oral therapy on an outpatient basis. Many patients with tularemia, however, are gravely ill; because of high mortality rates, such patients warrant inpatient hospitalization for treatment with parenteral aminoglycosides 2.

## Outcome and Follow‐up

The patient improved rapidly after she began taking ciprofloxacin. Her splenomegaly resolved, her lower extremity ulcer healed fully and her myalgia, fatigue, and dyspnea abated. She was asymptomatic at a follow‐up appointment 2 months after the onset of her symptoms.

## Discussion

Tularemia remains a rare diagnosis worldwide. In the United States, the highest incidence is seen in the south central Midwest, the Pacific Northwest, and Massachusetts 3. Typically, tularemia is transmitted via arthropod vectors or by direct contact with or ingestion of rabbits, hares, or other small rodents infected with the disease. Rarely, birds, sheep, and even domesticated animals such as cats and dogs can be infected. Geographic variation in prevalence as well as the number of potential vectors for tularemia transmission highlights the importance of taking a complete exposure and travel history when considering this diagnosis. When injected or inhaled, as few as ten organisms can comprise an infectious dose. Due to the high degree of infectiousness of the inhaled form, tularemia is a reportable disease and classified as a Class A bioterrorism agent in the United States 4.

Clinical manifestations of tularemia typically occur between 3 and 5 days following infection. While some have proposed a binary taxonomy for categorizing tularemia syndromes, the clinical manifestations are classically and most commonly grouped into six categories. These categories include ulceroglandular, glandular, oculoglandular, pharyngeal, pneumonic, and typhoidal tularemia. Site of inoculation, tularemia strain and infectious dose determine the clinical manifestations of the disease 5. Ulceroglandular tularemia, the most common presentation, is characterized by fever, chills, and myalgia; an ulcerating, painful, maculopapular lesion at the site of inoculation; and regional lymphadenopathy. Glandular tularemia shares many of the same characteristics as ulceroglandular but lacks skin involvement. Oculoglandular and pharyngeal forms are caused by direct inoculation with infected material or by ingesting undercooked infected meat. Tularemia pneumonia occurs when bacteria are ingested or inhaled. Pneumonic forms can be seen in combination with typhoidal forms and both are associated with higher mortality. Recent outbreaks in the United States have been notable for higher rates of pneumonic tularemia 5. Typhoidal manifestations lack skin and lymph node involvement and can be difficult to diagnose given the lack of localizing signs and symptoms. In typhoidal presentations, patients typically experience fever, chills, myalgias, and arthralgias and may demonstrate splenomegaly or hepatomegaly. Typhoidal tularemia is also associated with higher mortality particularly in patients with significant comorbidities 2. There are reported cases of overlapping tularemia syndromes 6, 7, as in this case of ulceroglandular tularemia complicated by splenomegaly. Additionally, case reports underscore the difficulty of distinguishing tularemia from malignancy given the degree of lymphadenopathy that can accompany the infection 8, 9.

The diagnosis of tularemia is most often confirmed when there is a fourfold or greater increase in antibody titers in two blood samples drawn between one and four weeks apart. False‐positive serologic tests can be due to cross‐reactive antibodies to *Brucella*,* Legionella*, and other gram‐negative bacteria, specifically *Yersinia* and *Proteus* 10, 11.

In the case presented, the patient's symptoms were initially attributed to a spider bite. This is not an uncommon pitfall in the diagnosis of tularemia as it is often mistaken for other more common entities 12, 13, 14. This case highlights the importance of including tularemia on the differential diagnosis of a tender skin ulcer with associated adenopathy. Fortunately in this case, the dramatic degree of elevation of the patient's *F. tularensis* antibody titer (1:1280) was deemed sufficient for diagnosis, and unlikely to represent a false‐positive, in the context of her signs, symptoms, and epidemiologic risk for infection. Following diagnosis, the patient did reveal that she had a wild rabbit den in her backyard that was likely the source of her infection.

Clinicians should be aware that in North America and Europe, the only substantiated cause of necrotic spider bites are *loxosceles* spiders. In the United States, these spiders can be found in the Midwest and along the United States/Mexico border. Lesions caused by *loxosceles* are typically self‐limited and require no treatment other than local wound care. The true danger in loxoscelism stems from misdiagnosis and resultant delay in proper treatment of entities that, unlike spider bites, are not self‐limited and self‐healing. Providers should educate themselves on biting insects and spiders that are endemic to their region so as to avoid misdiagnosis.

## Authorship

HS: drafting of the article, critical revision of the manuscript, final approval. MM: critical revision of the manuscript, final approval. JC: critical revision of the manuscript, final approval.

## Conflict of Interest

None declared.
